# Silver and Hyaluronic Acid-Coated Gold Nanoparticles Modulate the Metabolism of a Model Human Gut Bacterium *Lactobacillus casei*

**DOI:** 10.3390/nano12193377

**Published:** 2022-09-27

**Authors:** Wenqian Huang, Yirong Zhang, Zhi Li, Minjie Li, Fangfang Li, Monika Mortimer, Liang-Hong Guo

**Affiliations:** 1 College of Life Science, China Jiliang University, Hangzhou 310018, China; 2 Institute of Environmental and Health Sciences, China Jiliang University, Hangzhou 310018, China; 3 College of Quality and Safety Engineering, China Jiliang University, Hangzhou 310018, China

**Keywords:** commensal bacteria, microbiota, bacteriocin, Caco-2, intestinal fluid, antibacterial

## Abstract

Medical applications of nanotechnology are promising in creating efficient and targeted therapies. However, so far, nanodrug design has not taken into consideration possible effects on human microbiota. The beneficial functions of bacteria could be stimulated by nanodrugs while negative effects on beneficial bacteria could cause risks to human health. Here, simulated intestinal fluid (IF) was optimized for culturing a human commensal and probiotic bacterial strain, *Lactobacillus casei*, to study the effects of medically relevant NPs—Ag and hyaluronic acid-coated Au NPs (HA-Au NPs)—in conditions pertinent to the gastrointestinal tract. When cultivated either aerobically or anaerobically, the specific growth rates of *L. casei* were ~0.2 h^−1^ in IF and ~0.4 h^−1^ in the standard medium of lactobacilli (MRS). Ag NPs inhibited the growth of *L. casei* in IF at lower concentrations (EC_50_ ~ 65 and 15 mg/L in aerobic and anaerobic conditions, respectively) than in MRS (EC_50_ > 100 mg/L), likely caused by differences in the composition of the two media and different intrinsic growth rates of bacteria in IF and MRS. Ag NP dissolution in IF and MRS did not explain the differences in growth inhibition, implying NP-specific effects. HA-Au NPs were not growth-inhibitory to *L. casei* up to 250 mg/L. Still, both NPs at sub-growth-inhibitory concentrations suppressed the expression of bacteriocin genes in *L. casei*, suggesting an inhibitory effect of NPs on the probiotic properties of *L. casei*, i.e., its competitiveness in microbial communities. However, HA-Au NPs did not appear to affect or even stimulated the immunomodulatory properties of *L. casei* in human intestinal epithelial cells. Thus, medically relevant NPs at low, sub-bacteriostatic levels can affect the metabolism of beneficial human bacteria and potentially induce changes in the microbiota and immune signaling.

## 1. Introduction

Engineered nanomaterials are studied as promising agents to treat diseases like cancer, neurological conditions and gastrointestinal diseases [1]. The global nanomedicine market has been estimated to reach $414 billion by 2027 [2]. Currently, there are more than 75 US FDA-approved nanomedicines in the market and over 100 in the clinical trial phase [3]. The advantages of using nanomaterials in the biomedical field include properties such as small size (~10–100 nm), large specific surface area and tunable surface coating which enable targeted delivery of therapeutics, improved solubility, bioavailability and reduced systemic toxicity of conventional drugs. Specific coatings and functionalization of nanoparticles (NPs) are used to improve the targeted delivery of the drug cargo, enhance NP surface hydrophilicity, evade uptake by phagocytes, prolong drug retention, and create pH- and hypoxia-responsive nanomedicines [4].

The most commonly used inorganic NPs in nanomedicine design include Au NPs as biocompatible drug carriers and photothermal therapy agents due to strong surface plasmon resonance (SPR). For targeted drug delivery, NPs are often functionalized with hyaluronic acid (HA) because of their affinity to the cell surface glycoprotein CD44 receptors, overexpressed in cancer cells [5]. Recently, HA-bilirubin nanoconjugate was proposed as an orally administered targeted therapy for inflammatory bowel disease (IBD) [6]. The study showed that in addition to targeting the inflamed colonic epithelium via HA interaction with CD44 receptors, the therapeutic efficacy of HA-bilirubin NPs was also mediated by changes in the gut microbiome, but the exact mechanisms remained unclear. Also, Ag NP-based therapies are often designed to combat bacterial infections, due to the excellent antimicrobial properties of Ag NPs [7], but also to target cancer cells [8]. Nanomedicines that target colon cancer or IBD will inevitably result in NP interactions with the gut microbiota. In addition, many nanomedicines are eliminated from the body via the gastrointestinal tract where NPs come into contact with commensal microorganisms. Research using in vitro and in vivo models has shown that NPs can disturb gut homeostasis by interacting with the intestinal epithelium as well as with microbiota [9]. For example, studies with mice and rats exposed to Ag NPs, which are the most widely used antibacterial NPs [7], indicated that Ag NPs induced gut dysbiosis and disturbed the homeostasis of the gut bacteria. Ag NPs decreased the abundance of phylum *Firmicutes*, which includes several probiotic and immunoprotective bacterial taxa, in the murine guts, which coincided with the negative effects on the murine intestinal and behavioral health [10,11]. Also, certain capping or coating agents used to stabilize Au NPs, such as citrate, polyvinylpyrrolidone (PVP), or tannic acid have been shown to induce gut dysbiosis in vivo [12]; these studies underscore the importance of considering NP effects on human microbiota in nanomedicine design and safety assessment.

In the complex bacterial community of the gastrointestinal tract, each strain colonizes its niche by synthesizing metabolites that support their colonization, nutrient uptake and help eradicate competitive bacterial strains, including pathogens [13]. In addition, commensal bacteria stimulate human immunoprotective functions and act as probiotics. For example, *Lactobacillus casei*, a probiotic commensal bacterium that modulates mucosal innate responses, has been shown to downregulate the genes encoding pro-inflammatory effectors such as cytokines, chemokines and adherence molecules induced by an invasive pathogen *Shigella flexneri* [14]. The immunomodulatory and antibacterial properties of *L. casei* were also shown to play a role in the protection of colon cancer development in an in vivo murine model [13]. It has been established that NPs can modulate the composition, abundance and richness of the commensal gut microbiota, disrupting the healthy microbial balance in the host [9]. It is also known that the crosstalk between the microorganisms and epithelial cells is an integral part of the immune response of the host [15]. Local immune homeostasis is maintained by epithelial cell signaling triggered by microbial components. Relatively less well established to date are the potential consequences of NP exposures to the crosstalk between the epithelial cells and commensal microbiota and to the ability of the beneficial bacteria to fight infections by pathogens. The effects are plausible because NPs at sub-bacteriostatic concentrations can affect important bacterial pathways such as energy metabolism, membrane transport and signal transduction, quorum sensing, as well as biofilm formation [16,17]; these important functions are crucial for the survival and thriving of bacteria in a healthy microbiome. Understanding how NPs regulate the metabolism of human commensal bacteria and pathogens at the molecular level provides new opportunities for developing innovative treatments for human disease.

Since the safety and efficacy of nanomedicines are often obstacles in their clinical translation [18], the mechanisms of action, biotransformation and fate of engineered nanomaterials after interacting with the intestinal microbiota and epithelial cell-microbiota crosstalk need to be understood. Here, *L. casei*, a human commensal and probiotic bacterial strain was used as a model commensal gut bacterium to study the effects of medically relevant NPs—Ag NPs and HA-coated Au NPs (HA-Au NP) on bacterial growth, competitiveness (bacteriocin transcription) and immunomodulatory properties in human intestinal epithelial cells (Scheme S1). Simulated intestinal fluid (IF) was formulated to support the growth of *L. casei* for NP effect assessment in physiologically relevant conditions and NP biotransformations were characterized in IF containing bacterial secreted metabolites. The results of this model system-based study guide the future design of nanomedicine safety evaluation studies in physiologically relevant conditions taking into consideration the potential microbiota-mediated human health effects.

## 2. Materials and Methods

### 2.1. Preparation of Ag NP Dispersion and Synthesis of HA-Au NPs

Uncoated Ag NPs (60–80 nm, XFJ14-1) were purchased as a powder from XFNano (Nanjing, China). Ag NP stock in ultrapure water (resistivity of 18.2 MΩ cm^−1^, Direct-Q system Millipore, Billerica, MA, USA) was prepared by dispersing 100 mg of NP powder in 50 mL of water in a sterile, acid-cleaned glass bottle. The NP dispersion was bath-sonicated for 15 min and stored at 4 °C. Before each use, the stock dispersion was bath-sonicated for 15 min.

HA-Au NPs were prepared via the reduction of gold ions (Au^3+^) by NaBH_4_ in water, according to the procedure reported previously [19]. Briefly, HAuCl_4_ (4.8 mL, 10 mg/mL, powder purchased from Aladdin Biochemical Technology Co., Shanghai, China) and HA sodium salt solution (25 mL, 0.1 mg/mL, HA sodium salt powder, MW 799.64, was from Energy Chemical, Shanghai, China) were mixed and stirred (250 revolutions per minute or rpm) for 3 min. Then, NaBH_4_ (1 mL, 4 mg/mL) was added to the mixture while stirring. The stock solution gradually changed from yellow to red-purple, indicating the formation of HA-Au NPs. Finally, the as-prepared HA-Au NPs (in total 30.8 mL) were loaded into a 1-kDa molecular weight cutoff (MWCO) dialysis membrane (regenerated cellulose, diameter 24 mm, Spectra/Por^®^6 Dialysis Membranes, Sangon Biotech, Shanghai, China), placed into 1.5 L of ultrapure water and dialyzed on a magnetic stir plate (150 rpm) for 24 h. Water was changed every 3 h. After dialysis, the purified HA-Au NPs in ultrapure water were transferred to a sterile tube and stored at 4 °C. The concentration of HA-Au NPs stock was determined by inductively coupled plasma-mass spectroscopy (ICP-MS) as described below.

### 2.2. Characterization of NPs 

Scanning electron microscopy (SEM) and transmission electron microscopy (TEM) were used to characterize the morphology and pristine size of Ag NPs and HA-Au NPs. For SEM, 2 µL of Ag NP (2 g/L) or HA-Au NP (1 g/L) stock dispersions were pipetted on a conductive carbon adhesive tape. Excess water was removed with filter paper, the samples were air-dried and then imaged using HITACHI SU8010 SEM, Tokyo, Japan. For TEM, 2 µL of Ag NP or HA-Au NP stock dispersions were diluted to 0.2 g/L. Then, 2 µL of the NP dilution was pipetted on a carbon film supported by a copper grid, air-dried and imaged with HITACHI HT7700 TEM, Tokyo, Japan. NP size distributions were measured from the TEM and SEM images using ImageJ [20]. Approximately 100–200 particles were measured for each NP type. The mean hydrodynamic diameters (HDD) of Ag NPs and HA-Au NPs in water or medium were determined by dynamic light scattering (DLS) measurements using a Zetasizer Nano S90 instrument (Malvern, UK). The ζ potential of NPs was determined with nanoPartica SZ-100V2 (Horiba, Kyoto, Japan). For the measurements, NPs were diluted to 100 mg/L in the respective media and immediately measured in triplicates. Ultraviolet-visible (UV-Vis) absorption spectra were recorded on a Shimadzu UV-Vis spectrophotometer (UV-3600 Plus, Tokyo, Japan). Ag NP spectra were measured in the range of 300–700 nm and HA-Au NP spectra between 400–800 nm.

### 2.3. Bacterial Strains, Growth Media and Culturing

*L. casei* (CGMCC 1.2435) culture was obtained from the China General Microbiological Culture Collection Center (CGMCC, Beijing, China). The strain was stored at −80 °C in 15% (*v*/*v*) glycerol in de Man, Rogosa, and Sharpe (MRS) broth (Aladdin, Shanghai, China). For experiments, *L. casei* from the frozen stock was streaked onto MRS agar and cultivated at 37 °C for two days, aerobically. The liquid media used for culturing *L. casei* were MRS broth and simulated intestinal fluid (IF). IF was formulated to closely simulate physiological conditions and simultaneously support the growth of *L. casei* [21,22]. The final optimized composition of IF was: 6 g/L CH_3_COONa, 1 g/L K_2_HPO_4_, 0.01 g/L NaCl, 1.0 g/L MgCl_2_ × 6H_2_O, 0.01 g/L MnSO_4_ × H_2_O, 1.1 g/L Tween 80, 0.1 g/L bile salt, 10 g/L glucose (all from J&K Scientific, Beijing, China) and 10.4 g/L meat extract (Solarbio, Beijing, China). Glucose and meat extract were added as a representative meal and to support bacterial growth. 100 U/mL of trypsin (J&K Scientific, Beijing, China) was added to the autoclaved medium immediately prior to experiments. For culturing in MRS or IF, 5–7 colonies of *L. casei* were first transferred from MRS agar to liquid MRS and cultured at 37 °C, without shaking, aerobically. The liquid cultures in the late-exponential phase, i.e., optical density at 600 nm (OD_600_) ~0.7, were harvested by centrifugation at 5000× *g* for 5 min and resuspended in IF or MRS. The bacterial cell suspension was adjusted with fresh MRS or IF to OD_600_ 0.01 and 100 µL was pipetted into the wells of a polypropylene 96-well clear plate (NEST, Wuxi, China). Wells containing 100 µL of MRS or IF were used as medium controls. The microwell plates were incubated at 37 °C and the OD_600_ at every hour was recoded using a Spark microplate reader (TECAN, Männedorf, Switzerland). To achieve anaerobic conditions, 100 µL of sterile mineral oil (Sigma-Aldrich, St. Louis, MO, USA) was pipetted onto the bacterial suspensions and medium controls in the microplate wells, as reported previously [23]. The pH of the bacterial growth medium during culturing was measured with indicator paper. For this, portions of cultures were transferred to 1.5-mL centrifuge tubes, and bacteria were separated from the media by centrifugation at 10,000× *g*, 10 min. Bacterial strain *Micrococcus luteus* (CGMCC1.3749, Beijing, China) was maintained as a frozen stock at −80 °C in 15% (*v*/*v*) glycerol in Nutrient Broth. Before experiments, frozen bacteria were streaked onto Columbia Blood agar (CBA, consisting of Columbia agar base, from Oxoid, supplemented with 5% sheep blood, from Nanjing Lezhen Biotechnology Co., China, and 0.1% CaCO_3_) and cultured for 48 h at 37 °C, aerobically. *M. luteus* was used as the bacteriocin-sensitive indicator organism in the antibacterial activity test.

### 2.4. Bacterial Growth Assays with NPs

For NP exposure experiments, bacteria were grown with and without Ag or HA-Au NPs in polypropylene 96-well clear plates (NEST, Wuxi, China). Portions of NP stocks in water were initially mixed at a 1:1 ratio with a twice concentrated culture medium and then diluted further with MRS or IF to prepare NP dispersions at twice the final exposure concentrations. For bacterial growth assays, 50 µL of each NP dilution was pipetted into the wells of a 96-well microplate and inoculated with 50 µL of bacteria in MRS or IF. The OD_600_ of bacterial inoculum was ~0.02, to yield the starting OD_600_ ~0.01 in the microplate wells. The exposure concentrations of Ag NPs were 0.1, 1, 10, 100 mg/L and concentrations of HA-Au NPs were 12.5, 25, 50, 250 mg/L. AgNO_3_ was used as a positive control at 625 or 100 mg/L in MRS and IF, respectively, which inhibited bacterial growth by ~50–80% compared to the control bacteria. Wells with only media were included as uninoculated controls and wells with bacteria in media without NPs as control bacteria. The plates were incubated at 37 °C without shaking. To quantify bacterial growth, the contents of two replicate wells from each treatment and control were sacrificed every two hours for ATP measurement. For that, an ice-cold ATP extraction solution consisting of 4% trichloro-acetic acid (TCA) and 4 mM ethylenediaminetetraacetic acid (EDTA) was added to the microplate wells in a 1:1 ratio with the bacterial culture. After mixing by pipetting the samples were transferred to 1.5-mL centrifuge tubes. The tubes were vortexed and placed in ice for 10 min before transferring to −20 °C until analysis. In microplates where the wells contained layers of mineral oil to achieve anaerobic conditions, the oil layer was removed by pipetting before adding ATP extraction solution. ATP was quantified using ATP Bioluminescent Assay Kit (Sigma-Aldrich, St. Louis, MO, USA) as reported previously [24]. Specific growth rates were calculated to construct dose-response curves and to model the EC_50_ values (the effective concentration that induces a response in 50% of the population). EC_50_ values were obtained using REGTOX macro for Microsoft Excel™ [25].

### 2.5. RNA Isolation and Quantitative PCR Analysis

The expression of bacteriocin genes of *L. casei* was quantified by using real-time quantitative polymerase chain reaction (qPCR) analysis. *L. casei* inoculum was prepared as described above and used to inoculate 3 mL of MRS broth or IF that contained Ag NPs at 0.1 mg/L or HA-Au NPs at 12.5 or 25 mg/L in a 24-well plate (NEST, Wuxi, China). The initial cell concentration was adjusted to OD_600_ 0.01. Bacterial cultures with or without NPs were grown for 16 h in MRS broth or 40 h in IF, at 37 °C anaerobically. Oxygen was depleted by placing the plates in a sealed box containing AnaeroPack-Anaero sachets (MGC, Niigata, Japan). At the late exponential phase the cultures were pipetted into centrifuge tubes and centrifuged at 5000× *g* for 10 min at 4 °C to harvest *L. casei*. To extract RNA, bacterial cells were suspended in 1 mL Trizol reagent (Takara Bio, Kusatsu, Japan), mixed by pipetting, and kept at room temperature for 5 min. Then chloroform was added at 0.2 times the sample volume, mixed vigorously for 15 s, kept at room temperature for 2–3 min and centrifuged at 12,000× *g*, 4 °C for 15 min. The upper aqueous phase (600 µL) containing RNA was transferred to a new centrifuge tube, where RNA was precipitated by adding an equal volume of isopropanol, vigorous shaking and incubation at 4 °C for 10 min. RNA was pelleted by centrifugation at 12,000× *g*, 4 °C for 10 min and washed with 700 µL of 80% ethanol, centrifuged at 12,000× *g*, 4 °C for 5 min and dried (5–10 min). Then, RNA was dissolved in 15 µL of diethyl pyrocarbonate (DEPC)-treated water and the concentration was quantified by using a spectrophotometer for nucleic acid and protein quantification (Nanoready, Aosheng Instruments, Hangzhou, China). PCR primers of *L. casei* ATCC334 (CGMCC 1.2435) bacteriocin genes were selected from published literature (Table 1) [26]. Glyceraldehyde-3-phosphate dehydrogenase (GAPD) was used as a reference gene. SYBR Green Master Mix (Yeasen, Shanghai, China) was used for the qPCR reaction with a total volume of 20 µL, consisting of 1 µL cDNA template, 10 µL SYBR Green Master Mix, 0.5 µL each of forward and reverse primers (10 µM), and 8 µL of ultrapure water. The qPCR reaction conditions were as follows: 95 °C for 5 min, followed by 40 cycles of 95 °C for 10 s, 60 °C for 30 s, and 72 °C for 20 s. The qPCR analysis was conducted in three technical replicates. The results were analyzed using the comparative Ct method (2^(−ΔΔCt)^) [27].

### 2.6. Preparation of Spent Media and Their Antibacterial Activity Testing

Approximately 5–7 *L. casei* colonies from the MRS agar plate were transferred into a liquid growth medium, either IF or MRS, and cultivated anaerobically for 48 or 24 h, respectively. Bacteria were pelleted by centrifugation (10,000× *g*, 4 °C, 10 min), the supernatant was collected, passed through a 0.45 µm filter (Jet Biofil, Guangzhou, China) and freeze-dried. The antimicrobial activity of the spent media was assessed using the deferred antagonism assay [28]. Freeze-dried spent media from 20 mL of original volume were resuspended in 500 µL of sterile ultrapure water, 5 µL was spotted onto the CBA plate and allowed to sorb into the agar. Then, 3 mL of 0.5% (*w*/*v*) Nutrient Broth agar at 50 °C was inoculated with indicator bacteria *M. luteus*. *M. luteus* suspension was prepared by suspending several colonies from CBA agar into Nutrient Broth to achieve OD_600_ ~ 0.6, then 100 µL of inoculum was pipetted into 3 mL of warm agar. The inoculated agar was then poured onto the CBA plate with spotted spent media from *L. casei*. Nisin (100 IU/mL, Sigma Aldrich, St. Louis, MO, USA) spotted on the CBA agar was used as a positive control. Agar plates were incubated overnight at 37 °C aerobically. The diameters of inhibition zones on agar were measured to quantify the antibacterial activity of *L. casei* spent media.

### 2.7. Inductively Coupled Plasma-Mass Spectroscopy (ICP-MS)

To determine the concentration of Au in the synthesized HA-Au dispersion, 200 µL of HA-Au dispersion was mixed with 800 µL of aqua regia. After keeping the samples for 24 h at room temperature, they were diluted with ultrapure H_2_O for analysis. To quantify the release of Ag^+^ and Au^3+^ from Ag and HA-Au NPs, respectively, NPs were dispersed in fresh MRS or IF for immediate measurement (0 h timepoint) or in spent MRS or IF and incubated for 24 h at 37 °C to simulate *L. casei* exposures to NPs. Specifically, NPs were dispersed at 0.1, 1, 10 or 100 mg/L for Ag NPs and at 12.5, 25, 50 or 250 mg/L for HA-Au NPs in media in a 24-well plate with a volume of 1.2 mL per sample. Three replicate wells were set up per sample. The plates were incubated at 37 °C either aerobically or anaerobically in a sealed box with AnaeroPack-Anaero sachets, for 24 h. The collected samples were centrifuged at 15,000× *g*, 4 °C, for 30 min. Then, the top 200 µL of the supernatant was collected and mixed with 2 mL of 5% nitric acid. NP samples in fresh media were prepared similarly as in spent media, but immediately centrifuged and sampled for ICP-MS. The ICP-MS (iCAP RQTM, Thermo Scientific™, Waltham, MA, USA) linear range for Ag was 0.01–100 ng/mL and for Au 0.01–50 ng/mL. The limits of detection (LOD) of Ag and Au were 0.0022 and 0.004 ng/mL, respectively.

### 2.8. Characterization of NPs Incubated in Spent Media

To assess the transformation of NPs in the media that contained bacterially secreted compounds, NP stocks (Ag NPs at 2 g/L and HA-Au NPs at 1 g/L) were suspended in the spent media of *L. casei* (freshly collected after bacterial growth as described in Section 2.6). Specifically, 0.5 mL of NP stock was added to 9.5 mL medium in borosilicate test tubes and incubated at 37 °C, 200 rpm for 24 h, aerobically. After incubation, the hydrodynamic diameters of NPs were determined using a Zetasizer S90 instrument. The UV-vis absorption spectra were recorded on a Shimadzu UV-vis spectrophotometer. The concentration of Ag NPs was 50 mg/L and HA-Au NPs 25 mg/L when measuring the UV-Vis absorption. Protein adsorption on the NPs was determined by sodium dodecyl sulfate-polyacrylamide gel electrophoresis (SDS-PAGE). NPs were incubated in spent or fresh MRS or IF as described above, and NPs were collected by centrifugation at 10,000× *g* for 10 min and washed with ice-cold phosphate-buffered saline (PBS, pH7.5) once. Then, NPs were suspended in 20 µL of RIPA buffer and 5 µL of 5×SDS-PAGE sample loading buffer (Biosharp, Hefei, China) and heated for 10 min at 100 °C. After centrifugation at 12,000× *g* for 15 min, 4 °C, 10 µL of supernatant was transferred to SDS-PAGE gel (consisting of 5% stacking and 7.5% separating gel). The SDS-PAGE gel was run at 60 V for about 1 h, then at 140 V for about 40 min. The running buffers included a cathodic electrophoresis solution: 6.05 g Tris-base, 8.95 g Tricine, 5 mL 10% SDS added into 495 mL H_2_O, and an anodic electrophoresis solution: 12.12 g Tris-base, 1.66 mL HCl added into 500 mL H_2_O. Molecular weight standard covering the size range of 10–180 kDa (PageRuler Prestained Protein Ladder, Thermo Scientific, Waltham, MA, USA) was run in parallel with samples. The SDS-PAGE gel was stained with Coomassie Brilliant Blue R-250 (BioFroxx, Einhausen, Germany) to visualize the protein bands and imaged with ChemiDoc XRS+ system (Bio-Rad, Shanghai, China). The protein band intensities were quantified using ImageJ Gel Analyzer function.

### 2.9. Caco-2 Cell Culture, Differentiation and Exposure to Lipopolysaccharide and Spent Media

Human epithelial cell line Caco-2 (ATCC, Manassas, VA, USA) was cultured in MEM (Gibco, Grand Island, NY, USA) supplemented with 20% fetal bovine serum (FBS), 100 IU/mL penicillin and 100 µg/mL streptomycin at 37 °C, 5% CO_2_. Cells were subcultured every 3–4 days. For differentiation, cells were trypsinized and resuspended in DMEM (Gibco) supplemented with 10% FBS and penicillin and streptomycin at final concentrations of 100 IU/mL and 100 µg/mL, respectively. The cell concentration was adjusted to a seeding density of 4 × 10^5^ cells/cm^2^ and transferred to 12-well plates with transwell polycarbonate membranes (12 mm, 0.4 µm pore size, 1.12 cm^2^, Labselect, Hefei, China). The cells were incubated at 37 °C, 5% CO_2_, for 21 days until differentiation, while the media were changed three times a week. To determine the monolayer integrity, transepithelial electrical resistance (TEER) was monitored using Millicell ERS-2 volt-ohmmeter (EMD Millipore, Billerica, MA, USA). Differentiated Caco-2 cells were exposed to 5 µg/mL lipopolysaccharide (LPS, L6143, BioXtra, Sigma-Aldrich, St. Louis, MO, USA) in the basolateral compartment. The apical side contained either culture medium or culture medium supplemented with spent MRS or spent MRS from *L. casei* cultivated with HA-Au NPs at 50 mg/L, prepared as described in Section 2.6. The culture medium used for exposure was DMEM without FBS or antibiotics. To prepare spent media in DMEM, freeze-dried spent media from 15 mL of original volume were resuspended in 150 µL of sterile ultrapure water, then diluted 100 times with DMEM. Control wells contained FBS- and antibiotic-free DMEM in both basolateral and apical compartments. Three replicate wells were set up for each treatment and control. The plates were incubated at 37 °C, 5% CO_2_, for 24 h. After exposure, Caco-2 cells were collected for RNA extraction and qPCR analysis. For that, media were removed from the wells and 400 µL of Trizol reagent (Takara Bio, Kusatsu, Japan) was added on the apical side of the membrane and mixed carefully but quickly by pipetting to avoid dissolving the membrane in phenol. The cell suspension was transferred into RNase-free tubes and stored at −80 °C. The expression of tumor necrosis factor alpha (TNF-α), prostaglandin-endoperoxide synthase 2 (PTGS2) and tight junction protein 1 (TJP1) were quantified by qPCR as described in Section 2.5. Glyceraldehyde-3-phosphate dehydrogenase (GAPDH) was used as a reference gene. The primers used are listed in Table S1.

### 2.10. Statistical Analysis

All experiments were performed in triplicate and the results were presented as mean ± standard deviation (SD). Statistical analysis was performed using Excel and GraphPad Prism. The differences between the groups were assessed by one-way ANOVA and Student’s *t*-test or Tukey’s multiple comparisons test. The results were considered statistically significantly different at *p* < 0.05.

## 3. Results and Discussion

### 3.1. Formulation of Simulated Intestinal Fluid for Culturing of L. casei

Lactobacilli are fastidious bacteria that require specifically formulated media for efficient cultivation. One of the most commonly used rich growth mediums optimized for lactobacilli is MRS, which was developed in the 1960s [29]. It contains peptone, yeast extract and beef extract as the main sources of nitrogen, amino acids, peptides, nucleic acids, minerals and vitamins, and glucose as a carbon source, while surfactants such as Tween 80 are added to protect cells in harsh conditions, improve nutrient uptake and serve as a source of oleic acids (Table 2). Mn^2+^ and Mg^2+^ are included as essential elements for the growth of lactobacilli, while Mn^2+^ also functions in the catalytic scavenging of oxygen, needed for anaerobic growth. In addition, buffering agents are necessary for balancing the pH during the growth of lactobacilli that produce acids during their metabolism of carbohydrates [30].

Here, to simulate physiologically relevant gastrointestinal conditions, while supporting the growth of *L. casei*, for the assessment of NP effects on the growth and metabolism of human beneficial lactic acid bacteria, simulated intestinal fluid (IF) was formulated. The starting point was the composition of simulated IF suggested for in vitro food and pharmaceutical digestion, which has been developed based on human in vivo IF parameters [22]. In this IF, the electrolyte and enzyme concentrations and the pH were set to simulate in vivo small intestinal conditions. The pH between the duodenum and distal ileum changes from ~6.5 to ~7.5, thus, the recommended pH of IF is 7. The digestion in the small intestine largely relies on pancreatic enzymes and bile, which are the recommended components in the simulated IF. Here, an individual proteolytic enzyme (trypsin at 100 U/mL) and bile salts were used. The function of bile in the intestine is the transport of lipolysis products, while lipolysis occurs more efficiently in the presence of bile. The suggested minimum IF composition was supplemented with nitrogen and carbon source (meat extract and glucose, respectively) to support bacterial growth (Table 2). Also, to satisfy specific requirements for the growth of lactobacilli, Tween 80, Mn^2+^ source and acetate were included [21]. It has been reported that Tween 80 increases the tolerance of bile salts by lactobacilli [31]. In addition, lactobacilli encode bile salt hydrolases that deconjugate bile acids, giving them advantages in colonizing the gastrointestinal tract as well as providing health benefits to the host by lowering cholesterol and producing signaling molecules [32].

**Figure 1:**
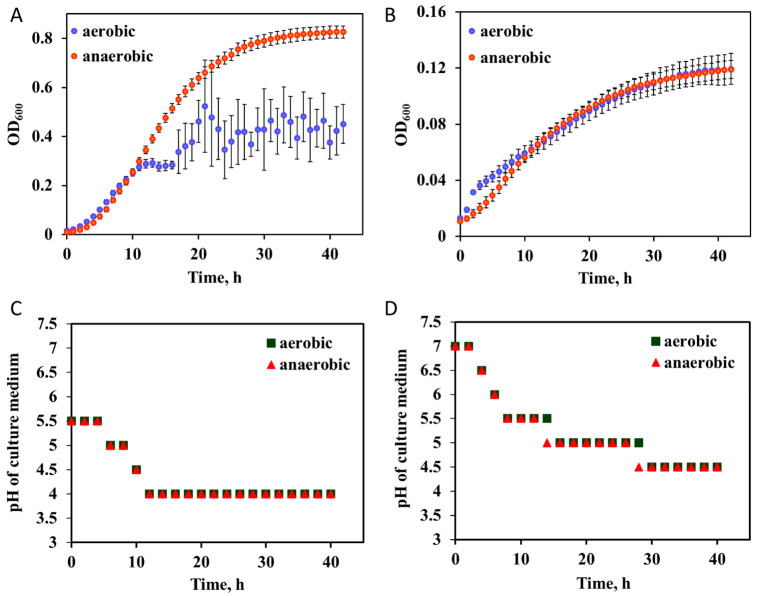
The growth of *L. casei* and accompanying changes in the pH of growth media in aerobic and anaerobic conditions. (**A**) Growth in the simulated intestinal fluid (IF), (**B**) growth in MRS, (**C**) pH of IF and (**D**) pH of MRS during *L. casei* cultivation. The data points are the average of 3 replicates and error bars indicate standard deviations. The error bars are not visible in panels (**C**,**D**) because the pH values measured with indicator papers did not vary between the replicates.

Growth parameters of *L. casei* in IF were established in parallel with cultures grown in MRS (the optimal growth medium for lactobacilli), in aerobic and anaerobic conditions. As a facultative anaerobe, *L. casei* is capable of respiratory metabolism in the presence of oxygen, while switching to fermentative metabolism in the anaerobic environment. In MRS, the growth of *L. casei* was similar in aerobic and anaerobic conditions until the cell concentration reached the OD_600_ ~0.3, with similar (*p* > 0.05) specific growth rates of 0.36 ± 0.01 and 0.4 ± 0.03 h^−1^, respectively (Figure 1A). However, in aerobic conditions, the exponential growth leveled off after reaching the OD_600_~0.3, while in anaerobic conditions the maximum growth yield was significantly higher at OD_600_~0.8; this characterizes well the physiological properties of lactobacilli which can tolerate O_2_ but due to the inability to synthesize heme molecules, their resistance to oxygen is limited; they have developed strategies, such as using zinc, manganese and selenium to scavenge reactive oxygen species (ROS), but tend to prefer fermentative rather than respiratory metabolism [33]. In IF, the specific growth rates in the aerobic and anaerobic environments were also similar (0.18 ± 0.04 and 0.20 ± 0.01 h^−1^, respectively, Figure 1B). However, they were significantly lower than the respective growth parameters in MRS. In addition, the maximum yields in IF were significantly lower than in MRS after 40 h cultivation but similar for the two oxygen conditions. The lower growth efficiency in IF than MRS is likely caused by the limited level of nitrogen, amino acid and vitamin sources provided in IF (only meat extract in IF compared to peptone, yeast and beef extract in MRS).

Another parameter that could have affected the growth yield was the acidification level of the growth media, due to lactic acid secretion of *L. casei*. The two media differed in their starting pH, with the pH of the commercial formulation of MRS after autoclaving being 5.5 and the pH of IF adjusted to 7 to mimic the conditions in the small intestine (Figure 1C,D). pH was monitored during bacterial growth in both media. By the mid-exponential growth phase, the pH of MRS had reached 4, while the pH of IF was initially rapidly lowered from 7 to 5.5 and then over a longer time frame reduced to 4.5. Thus, both growth media were similarly acidified by the stationary growth phase irrespective of the oxygen conditions. Overall, despite a lower specific growth rate and maximum yield compared to the rich growth medium, IF supported the growth of *L. casei* and thus could be used as a physiologically relevant exposure system to assess the effects of NPs on human beneficial gut bacteria.

### 3.2. Characteristics of Ag and HA-Au NPs and Their Transformation in Simulated Intestinal Fluid (IF)

**Figure 2:**
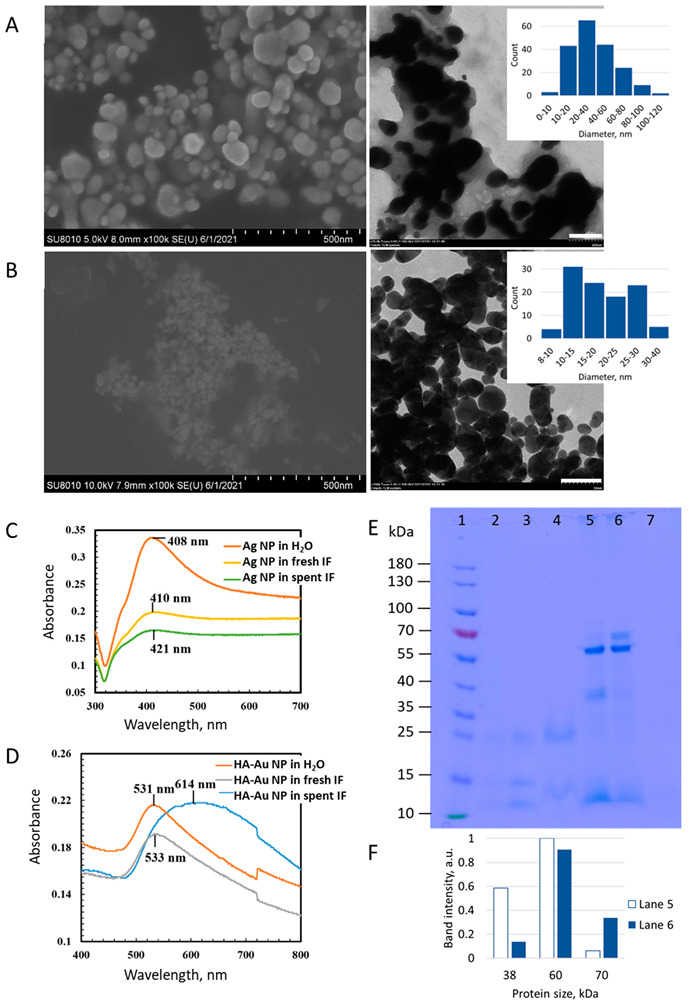
Characterization of NPs in pristine form, in water and growth media and after incubation in spent simulated intestinal fluid (IF). (**A**) SEM (scale bar = 500 nm) and TEM images (scale bar = 200 nm) and size distribution of Ag NPs, (**B**) SEM (scale bar = 500 nm) and TEM images (scale bar = 50 nm) and size distribution of HA-Au NPs, (**C**) UV-Vis spectra of Ag NPs in water, IF and spent IF, (**D**) UV-Vis spectra of HA-Au NPs in water, IF and spent IF, (**E**) SDS-PAGE analysis of proteins that were associated with NPs after 24-h incubation in fresh and spent IF: 1—protein ladder, 2—proteins associated with HA-Au NPs incubated in fresh IF, 3—proteins associated with Ag NPs incubated in fresh IF, 4—proteins in fresh IF, 5—proteins associated with HA-Au NPs incubated in spent IF, 6—proteins associated with Ag NPs incubated in spent IF, 7—proteins in spent IF, (**F**) quantitative analysis of protein bands in the SDS-PAGE gel, lanes 5 and 6 refer to lanes in panel (**E**).

Two commonly used medically relevant NPs were studied here for their effects on *L. casei* growth and metabolism. First, the NPs were characterized for their size and shape in pristine form and HDD and ζ-potential after dispersion in water and culture media. Electron microcopy images showed that Ag NPs had irregular morphology, with an average size of 39 ± 22 nm (Figure 2A), while HA-Au NPs were spherical with an average size of 19 ± 7 nm (Figure 2B); these sizes were comparable to the manufacturer-reported diameters of Ag NPs (60-80 nm) and the previously reported diameter of HA-Au NPs synthesized by using the same method as here [19]. TEM images also revealed a thin coating of hyaluronic acid on the surface of the HA-Au NPs. The DLS analysis showed that the average HDD of Ag NPs and HA-Au NPs in ultrapure water was expectedly larger than the pristine sizes (167 ± 20 nm and 55 ± 1 nm, respectively, Table 3) but the dispersions were stable as reflected by high absolute ζ-potential values of NPs and small HDD values after 24 h in water. HDDs of both NPs were significantly larger in the culture media compared to water, while Ag NPs agglomerated into larger particles than HA-Au NPs. Close-to-zero ζ-potential values also indicated the decreased dispersion stability of NPs in the media, likely promoted by high ionic strength of the media and potential coating of the NPs with media components [34]. While MRS is a complex rich medium, IF is a semi-synthetic medium containing meat extract, bile salts and trypsin, in addition to inorganic salts and glucose. Trypsin in IF is one of the three major human digestive proteases. Proteases, such as pepsin, which is another major protease secreted in the stomach, have been shown to promote the aggregation of Ag NPs by forming a protein coating around the NPs [35]. Trypsin may have played a role in NP agglomeration in IF, because the HA-Au NP dispersion stability was significantly reduced in simplified IF (i.e., no meat extract) that contained trypsin, compared to IF without trypsin (Figure S1). Lower OD_550_ values of HA-Au NPs in IF with trypsin compared to IF without trypsin indicated the sedimentation of agglomerated HA-Au NPs in the presence of trypsin, with agglomeration increasing over time. Still, considering that IF used in the study contained also meat extract, other proteins could have contributed to the agglomeration of NPs.

Since the degree of agglomeration and interactions with the culture media components can affect NP toxicological properties, NP transformations were assessed in spent IF (i.e., IF where *L. casei* was cultured for 48 h anaerobically and then bacteria were removed). While 24-h incubation of Ag NPs in fresh IF resulted in significantly higher agglomeration compared to 0 h in IF, there was no significant agglomeration of Ag NPs in spent IF after 24 h (Table 3), indicating the effects of bacterially secreted/consumed compounds on the physicochemical properties of NPs. HA-Au NPs, that were less prone to agglomeration in aqueous media than Ag NPs, due to surface-stabilizing HA molecules, did not show increased agglomeration during 24-h incubation either in fresh or spent IF and were better dispersed at ~200 nm size particles (compared to ~300 nm at 0 h, Table 3). Agglomeration of NPs in IF compared to water was also evident in the UV-Vis spectra (Figure 2C,D). The intensity of the characteristic Ag NP UV-Vis absorption peak at 408 nm was considerably reduced after 24-h incubation in fresh and spent IF, indicating increased agglomeration of NPs [36,37]. Also, the red-shift in the Ag NP peak suggested biomolecule coating acquired in IF. Similar to HDD measurements, UV-Vis spectra of HA-Au NPs indicated higher stability of HA-Au NPs than Ag NPs in IF: the intensities of the HA-Au NP characteristic absorption peaks due to surface plasmon resonance (SPR) at 531 nm and 533 nm [38] were similar in water and IF, respectively; this is consistent with the data reported for polyvinylpyrrolidone (PVP)-coated Au NPs which were stably dispersed in the simulated IF over 2 h, irrespective of the primary size of the NPs [39]. However, the absorption peak of HA-Au NPs after 24-h incubation in spent IF was red-shifted to 614 nm and had a broader shape compared to the peaks of HA-Au NPs in water or fresh IF (Figure 2D). Broadened absorption peak suggested HA-Au NP agglomeration and wider size distribution [40]. Due to the sensitivity of the SPR signal to both the adsorbed molecules on NPs and the aggregation state of NPs, the observed small shifts in the peak maxima indicate interactions of the NPs with biomolecules and larger shifts suggest NP aggregation.

The coating of NPs with proteins after incubation in spent IF was confirmed with SDS-PAGE (Figure 2E). Ag NPs and HA-Au NPs in spent IF (lanes 5 and 6 in the gel, respectively) adsorbed considerably higher amounts of proteins compared to NPs incubated in fresh IF (lanes 2 and 3, respectively). The adsorption of proteins to NPs in spent IF was evident from the strong protein bands compared to very weak bands in IF samples (lanes 4 and 7 for fresh and spent IF, respectively). Based on the SDS-PAGE gel, Ag NPs and HA-Au NPs adsorbed at least five different distinguishable proteins in spent IF, with molecular weights of ~12, 38, 60, 70 and 85 kDa. While both Ag and HA-Au NPs appeared to bind similar amounts of proteins with the size of ~60 kDa, as indicated by the strongest bands in the gel, there was a stronger band at ~70 kDa associated with HA-Au NPs than Ag NPs and the band at ~38 kDa was more pronounced in the case of Ag NPs (Figure 2F). Overall, Ag NPs seemed to be associated with a higher number of smaller proteins and HA-Au NPs with larger proteins. One of the factors that could determine protein binding to NPs is the size of NPs. For example, it was shown that smaller Ag NPs (size 20 nm) bound a considerably lower number of proteins than larger Ag NPs (110 nm) [41]. In addition, 20-nm sized Ag NPs were associated with a higher number of hydrophobic proteins than 110-nm Ag NPs, which suggested that NP curvature can determine the composition of protein coating on NPs. Here, the HDD of Ag NPs and HA-Au NPs were clearly different in spent IF (Table 3). Thus, NP size could have been a factor impacting the formation of protein corona around the NPs. Additionally, the surface properties of the two NPs differed and could have played a role in association with proteins. The protein sources in the simulated IF included trypsin (~23.3 kDa) and meat extract, which is an undefined complex mixture of proteins. Based on the literature, the main proteins in the meat extract include myosin heavy chain (~130 kDa) and light chains (~10–20 kDa), actin (~42 kDa), tropomyosin (~30 kDa) and desmin (53 kDa) [42]. During bacterial growth, some of the nutrients were consumed, and certain bacterial proteases and other proteinaceous biomolecules were secreted by the bacteria, but assuming the excess of nutrients in the growth medium, NP incubation in the spent medium likely resulted in the association of the meat extract proteins. The protein binding was clearly NP type-specific as indicated by different band intensities in the SDS-PAGE gel. However, some of the bacterially secreted compounds, such as bacteriocins, which are small peptides (3.5 kDa) [26], would not be resolvable in this gel and require a different experimental approach. Since NP coating with secreted biomolecules has been shown to impact the fate and cellular interactions of NPs [43], the identity and time evolution of protein and other biomolecule corona formation in physiologically relevant conditions, focusing on the bacterially secreted compounds deserves further study.

### 3.3. Ag and HA-Au NP Effects on the Growth of L. casei

The effects of Ag NPs and HA-Au NPs on the growth of *L. casei* were tested in parallel in the traditional rich growth medium for lactobacilli (MRS) and the simulated IF, to assess how the media composition affects NP antibacterial action. Also, the growth tests with and without NPs were conducted in aerobic and anaerobic conditions in both media to assess NP effects on bacteria with different metabolic activities (respiratory versus fermentative metabolism). The concentration ranges of Ag NPs and HA-Au NPs were selected based on published literature and the hypothesis that Ag NPs would be antibacterial while Au NPs would be inert. Thus, Ag NPs were tested at lower concentrations (0.1–100 mg/L) than HA-Au NPs (12.5–250 mg/L), while the maximum HA-Au NP concentration was limited by the turbidity/color interferences of the measurements.

**Figure 3:**
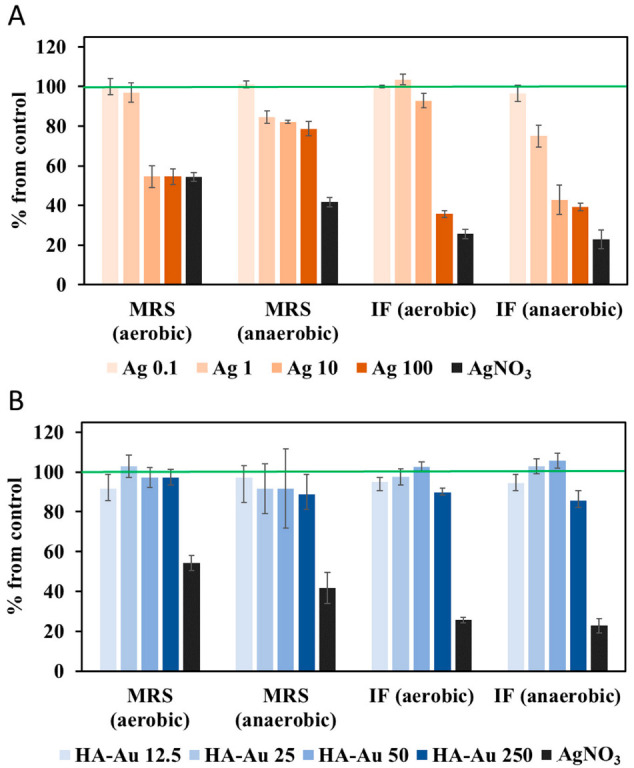
Effects of NPs on the growth of *L. casei* in different media and oxygen conditions. Relative specific growth rates compared to the unamended control culture (set as 100%, marked with green lines) in *L. casei* cultures (**A**) exposed to Ag NPs at 0.1, 1, 10 or 100 mg/L and (**B**) to HA-Au NPs at 12.5, 25, 50, 250 mg/L. AgNO_3_ was used as a positive control at 625 or 100 mg/L in MRS and IF, respectively, which inhibited bacterial growth by ~50% in MRS and ~80% in IF compared to the control bacteria.

Expectedly, Ag NPs were growth-inhibitory to *L. casei* (Figure 3A) due to the well-known antibacterial properties of Ag NPs [44,45]. Overall, *L. casei* was more sensitive to Ag NPs in IF than in MRS, both in aerobic and anaerobic conditions, based on the growth-inhibitory effects at 100 mg/L. While 50% growth inhibition was not reached in MRS at the tested concentration range of Ag NPs, the EC_50_ values calculated in IF were 65 and 15 mg/L in aerobic and anaerobic conditions, respectively; these relatively high effective concentrations of Ag NPs could be caused by media components (such as chlorides) that react with free Ag^+^ and make the antibacterial Ag species less bioavailable. For this reason, NaCl has been previously omitted from or reduced in the antibacterial tests of Ag NPs [46]. Still, in a study that used NaCl-free half-strength Luria Broth (LB), uncoated Ag NPs (with a primary size of 30–100 nm, comparable to the size of Ag NPs in this study) did not significantly inhibit the growth of different Gram-negative and Gram-positive bacteria at the maximum tested concentration of 40 mg/L [47]. Thus, the antibacterial concentrations of Ag NPs in this study are consistent with previous reports, and the relatively low toxicity could possibly be attributed to the large size of NPs. Indeed, smaller Ag NPs (10 nm) interacted with bacterial cells more efficiently than larger Ag NPs (~20–80 nm) and were shown to result in higher toxicity to bacteria [46]. Also, the comparison of differently sized citrate-coated Ag NPs indicated that smaller Ag NPs shed more Ag^+^ than larger Ag NPs resulting in higher toxicity of smaller NPs [45].

Another factor that could impact the sensitivity of bacteria to NPs is the specific growth rate which was significantly slower in IF compared to MRS (Figure 1A,B). Thus, there were fewer bacterial cells in IF at the same Ag NP concentration, resulting in a higher NP/bacterial cell ratio and possibly higher toxicity of NPs. However, since cell numbers changed over time during NP exposures, depending also on NP concentration, and the absolute cell counts were not determined in the current study, it was not feasible to estimate the NP exposure concentrations per bacterial cell in MRS or IF. Interestingly, also the toxicity of AgNO_3_ depended on the culture media; namely, a significantly lower concentration of AgNO_3_ was required to inhibit *L. casei* growth in IF (100 mg/L inhibited growth by ~80%) than in MRS (625 mg/L inhibited growth by ~50%); this could have been due to the lower intrinsic growth rate of bacteria in IF, but could have also been affected by the Ag ion speciation in the two media with different compositions. AgNO_3_ toxicity was not different in aerobic and anaerobic conditions, but Ag NP toxicity in MRS was more pronounced in aerobic than in the anaerobic environment (Figure 3A) which was expected due to oxidative dissolution of Ag NPs. Contrarily, in IF, Ag NP at 1 and 10 mg/L were more toxic in anaerobic than aerobic conditions which may be due to biocompound secretion by bacteria in IF and the resulting Ag NP dissolution.

**Figure 4:**
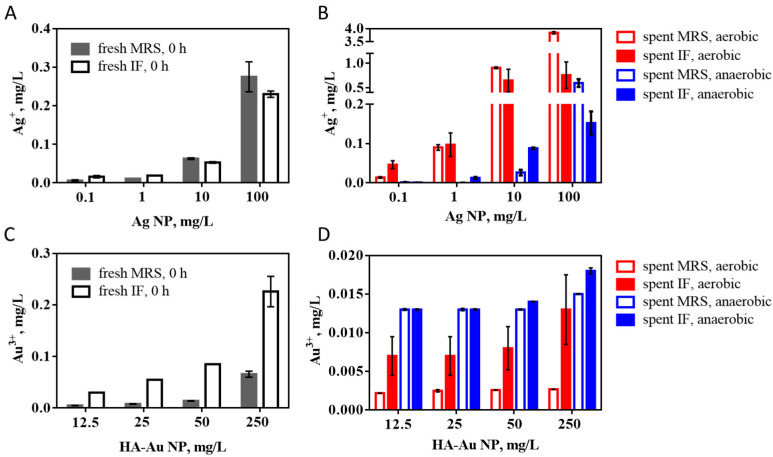
Dissolution of Ag NPs and HA-Au NPs in MRS and simulated intestinal fluid (IF). (**A**) Ag^+^ concentrations in MRS and IF immediately after diluting Ag NPs in the media, (**B**) Ag^+^ concentrations in spent MRS and spent IF after incubating Ag NPs in the media for 24 h, 37 °C, either in aerobic or anaerobic conditions, (**C**) Au^3+^ concentrations in MRS and IF immediately after diluting HA-Au NPs in the media, (**D**) Au^3+^ concentrations in spent MRS and spent IF after incubating HA-Au NPs in the media for 24 h, 37 °C, either in aerobic or anaerobic conditions.

To gain a better understanding of the silver speciation in the Ag NP dispersions in IF and MRS, dissolved Ag concentrations were quantified. Although there was no difference in the dissolution of Ag NPs in MRS and IF immediately after diluting in fresh media (Figure 4A), there were differences in the released Ag^+^ concentrations when Ag NPs were incubated in spent media (i.e., MRS and IF where bacteria were cultivated and then removed) either in aerobic or anaerobic conditions for 24 h at 37 °C (Figure 4B). It was evident that Ag NP dissolution was significantly higher in the aerobic environment than in anaerobic conditions in both media, while higher levels of Ag^+^ were released from Ag NPs (at 1 and 10 mg/L) in IF than MRS in anaerobic conditions. However, the concentrations of Ag^+^ in the spent media in any of the conditions or NP concentration did not reach the levels that would explain the growth inhibition of bacteria; the maximum dissolved concentration was ~3.8 mg/L Ag^+^ while bacterial growth was inhibited in the range of 100 and 625 mg/L AgNO_3_, corresponding to 63.5 and 397 mg/L Ag, respectively (Figure 3). Thus, the growth inhibition by Ag NPs must have been caused by the combination of NP and ion action. It is also possible that NP interactions with bacterial cell membranes induced higher Ag^+^ release than measured in spent media not containing bacteria [47].

HA-Au NPs were not significantly growth-inhibitory at any of the tested concentrations or conditions (Figure 3B). One plausible reason for this could be the negative surface charge of HA-Au NPs, due to electrostatic repulsion between the overall negatively charged bacterial cells and NPs. It has been experimentally confirmed previously that negatively charged Au NPs did not impact bacterial viability [48]. On the other hand, the levels of Au^3+^ released from HA-Au NPs in the growth media were insignificant, with the maximum at 0.23 mg/L in fresh IF at 0 h (Figure 4C); these concentrations were clearly below the potentially toxic levels of Au^3+^ to bacteria (growth-inhibitory EC_50_ reported at ~15–20 mg/L) [49]. The concentrations of free Au^3+^ after incubation of HA-Au NPs in spent media (pH ~4–4.5) for 24 h were one or two orders of magnitude lower than in fresh media (Figure 4D), even though low pH in biological matrices has been shown to stimulate dissolution of Au NPs [50]. Also, extracellularly secreted compounds were shown to mediate the dissolution of Au NPs [51]. However, the released gold ions are unstable and immediately associate with biological compounds that mediate the recrystallization of the dissolved gold [52]. Here, biomineralization could have occurred in the spent media, which would explain the significantly reduced Au^3+^ concentrations in spent media compared to fresh media immediately after diluting the HA-Au NP stock. Overall, the levels of metal ions released into the culture media either by Ag or HA-Au NPs were below growth-inhibitory concentrations to *L. casei*. Still, Ag NPs suppressed the bacterial growth in both media dose-dependently and the effects were dependent on the oxygen conditions as well as the media composition. Yet, in the same conditions, HA-Au NPs did not affect the growth of *L. casei*.

### 3.4. Ag and HA-Au NP Effects on the Bacteriocin Gene Expression of L. casei

In addition to the potential growth inhibitory effects of medically relevant NPs on the human beneficial bacteria, it is important to better understand NP impacts on the metabolism of bacteria due to the role of bacterial compounds in human digestion, immunity and signaling. Many different species of lactobacilli colonize the human intestine permanently, making up a considerable fraction (up to 1.8%) of the gut microbiota [33]. For survival and competitiveness in the complex environment of the intestinal microbiota, lactobacilli employ both cooperative and antagonistic strategies to interact with other microorganisms. One such antagonistic approach is the synthesis of antibacterial peptides called bacteriocins. Bacteriocins are ribosomally synthesized peptides with high specificity towards certain microbial species. *L. casei* genome has been found to encode eight bacteriocin-like peptides, while two of these have antibacterial activity against some lactobacilli and *Listeria* species [26]. Here, *L. casei* cultivated in MRS anaerobically for 24 h was confirmed to secrete antibacterial compounds in the culture medium. It was confirmed that the secreted compounds inhibited the growth of an indicator bacterial strain *M. luteus* (Figure S2). On the other hand, simulated IF where *L. casei* was cultivated for 48 h, did not show antibacterial activity. There could be several reasons for this: (i) the composition of simulated IF was not optimal for the induction of bacteriocin synthesis or the medium did not support sufficient growth to reach the culture densities required for bacteriocin production [53]; (ii) trypsin, a proteolytic digestive enzyme added to the IF, could have inactivated the secreted bacteriocins which are sensitive to trypsin [26]; (iii) bacteriocins were synthesized but were not released into the culture medium since some bacteriocin-producing lactobacilli adsorb bacteriocins on the cell membrane rather than secrete in the surrounding medium [54]; these aspects remain to be evaluated in future studies.

**Figure 5:**
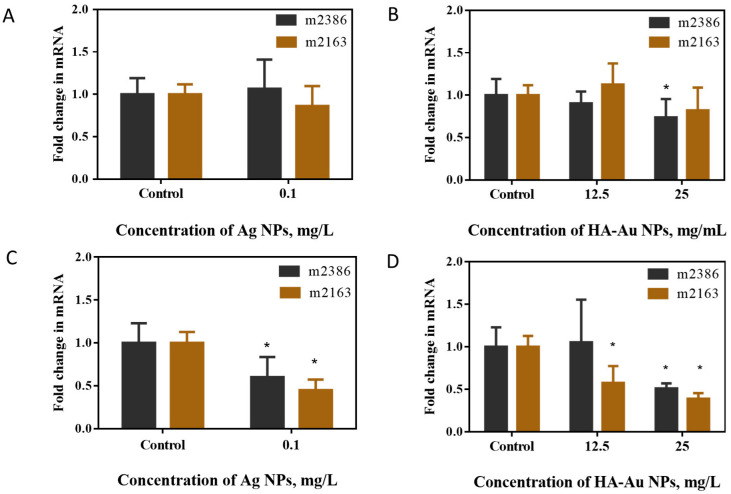
Bacteriocin gene expression in *L. casei* exposed to NPs at sub-growth-inhibitory concentrations compared to the gene expression in unamended control bacteria. Relative expression of genes (m2386 and m2163 for the peptides of LSEI_2386 and LSEI_2163, respectively) after exposure to (**A**) Ag NPs in simulated IF, (**B**) HA-Au NPs in simulated IF, (**C**) Ag NPs in MRS and (**D**) HA-Au NPs in MRS. * indicate significant differences between the control and NP treatments (*p* < 0.05, one-way ANOVA and Tukey’s multiple comparisons test).

Thus, to overcome these potential technical issues with measuring the antibacterial activity in IF, bacteriocin gene transcription was quantified to assess NP impacts on the antibacterial activity of *L. casei*. The two bacteriocin genes assessed were LSEI_2386 and LSEI_2163, which are cationic peptides consisting of 22 and 24 amino acids, respectively, and belong to the class II bacteriocin group [26]. After exposure of *L. casei* to Ag NPs and HA-Au NPs at sub-growth-inhibitory concentrations in IF for 40 h, anaerobically, there were overall no changes in bacteriocin gene expression (Figure 5A,B). The only statistically significant change was detected in the expression of LSEI_2386 after exposure to HA-Au NPs at a higher concentration. However, significant downregulation of both bacteriocin genes occurred upon exposure to NPs in MRS for 16 h (Figure 5C,D); this suggested that the bacterial metabolism had different susceptibilities to NPs in MRS and IF, which was likely connected to different growth profiles in the two media. Previous studies have shown that NPs can stimulate bacteriocin production in other species of lactic acid bacteria [55]; these studies have suggested that the stress induced by NPs can promote the secretion of antibacterial compounds, thus certain NPs could be potentially used as prebiotics [55,56]. However, here, bacteriocin gene transcription was inhibited by metal-based NPs in MRS while bacterial growth was not affected at these NP concentrations.

In *L. casei*, bacteriocin production is regulated by quorum sensing which involves constant secretion of low levels of an autoinducing peptide (LSEI_2386); this peptide then induces bacteriocin (i.e., LSEI_2163) expression through the two-component regulatory system, which involves a histidine kinase and a response regulator. Here, NPs at low concentrations did not reduce the bacterial cell numbers and thus, also quorum sensing would be unaffected. However, NPs could interact with bacterial cells and interfere with the signaling process which involves membrane-bound proteins. Previous studies with Gram-positive bacteria have shown that Ag NPs at sub-growth-inhibitory concentrations affect the production of quorum-sensing-related proteins [57]. Sub-lethal levels of Ag NPs have also been shown to affect proteins associated with biofilm formation of Gram-negative bacteria [58], suggesting interference with bacterial signaling and functional compound secretion. While low concentrations of certain types of metal NPs that contain biologically active metals (such as ZnO) can stimulate quorum sensing and biofilm formation via providing additional nutrients [59], Ag NPs as a non-essential metal NP may have a different effect. It is also possible that Ag NPs interfered with bacteriocin signaling by sequestering autoinducing peptides because Ag NPs have been shown to directly interact with bacterially secreted functional proteins to hinder biofilm formation [60]. Here we showed that Ag NPs adsorbed proteinaceous compounds in spent IF (Figure 2E), which likely also occurred in MRS and could explain Ag NP effects on bacteriocin gene expression.

The underlying mechanisms of HA-Au NP inhibitory effects on bacteriocin gene transcription are less clear. One of the strategies for targeting bacterial infections by HA-coating of NPs is activating the hyaluronidase production of bacteria to degrade HA around the NPs [61]. As a result, the NPs are stripped from the HA coating which reduces their dispersion stability. Reduced stability of Au NPs could result in the adsorption of NPs on bacteria and release of excess Au^3+^. However, no hyaluronidase-encoding genes have been identified in *L. casei* and hyaluronic acid has not been shown to inhibit the growth of lactobacilli [62]. Thus, the mechanisms of bacteriocin downregulation by HA-Au NPs in *L. casei* require further assessments. Nevertheless, although HA coating is expected to render Au NPs relatively well-dispersed in physiological fluids and biocompatible, the results of this study indicated that sub-growth-inhibitory levels of the medically relevant NPs can affect human beneficial microflora. Gene transcription-level changes that were measured in this study would need further investigation to quantify changes in gene products, i.e., bacteriocin peptides, to confirm the impacts of NPs on the antibacterial activity of *L. casei*.

### 3.5. HA-Au NP Effects on the Immunomodulatory Properties of L. casei

**Figure 6:**
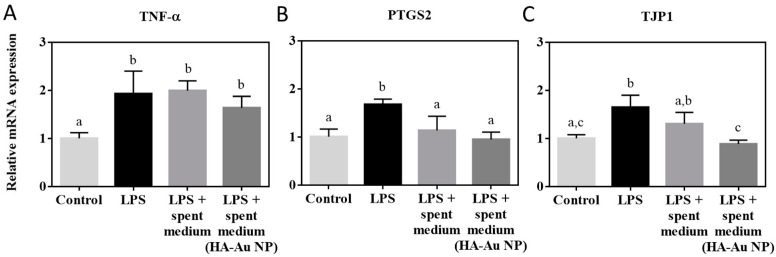
Relative mRNA expression in differentiated Caco-2 cells after exposure to LPS, LPS and *L. casei* spent medium or LPS and spent medium from *L. casei* exposed to HA-Au NPs. (**A**) Relative mRNA expression of TNF-α, (**B**) relative mRNA expression of PTGS2 or COX-2 and (**C**) relative mRNA expression of TJP1. Data bars are mean values (*n* = 3–6) and error bars indicate standard deviations, different letters indicate significant differences (*p* < 0.05, one-way ANOVA and Tukey’s multiple comparisons test).

In addition to having probiotic properties and fighting potentially pathogenic bacteria in the gut microbiota, *L. casei* also interacts with human cells directly and has immunoprotective effects. The beneficial effects of lactobacilli via regulating the signaling in human epithelial cells have been demonstrated [63]. For example, lactobacilli have been shown to modulate the transcription and synthesis of proinflammatory cytokines such as TNF-α and exert beneficial effects on colon histology in vitro and in vivo [64,65]. Thus, we next sought to explore if NP exposures could impact the anti-inflammatory properties of *L. casei*. Since the bacteriocin activity of *L. casei* was detected in the culture medium (MRS), we hypothesized that the spent MRS also contained immunomodulatory compounds. Thus, the impact of spent MRS was tested on the gene expression of differentiated human intestinal epithelial cells (Caco-2). Genes for inflammatory signaling (TNF-α, PTGS2 or COX-2) and tight junctions (TJP1) were used as markers of immunomodulation in Caco-2 cells. The expression of these genes was first stimulated by challenging differentiated Caco-2 monolayers from the basolateral side with LPS. As reported previously, LPS upregulated the expression of these genes significantly in Caco-2 cells [66] (Figure 6). However, when challenging Caco-2 cells with LPS simultaneously with added spent MRS, there was no significant upregulation of PTGS2 and TJP1 compared to unamended control cells, demonstrating the mitigating effect of *L. casei* secreted compounds. Nevertheless, *L. casei* spent medium did not suppress LPS-induced upregulation of TNF-α expression, indicating the lack or insufficient levels of bacterial compounds that would effectively counteract LPS-induced TNF-α stimulation (Figure 6A). The spent MRS medium obtained from *L. casei* incubated with HA-Au NPs exerted similar effects as the spent MRS of the unamended control bacteria (Figure 6A,B) or suppressed LPS-induced inflammation significantly more effectively (Figure 6C). It is worth noting that the possible effects of HA-Au NPs on the Caco-2 cells were excluded because the spent MRS was assumed not to contain any HA-Au NPs as these were removed during the preparation process of spent media (centrifugation steps and filter-sterilization). In addition, it has been reported previously that Au NPs did not affect the integrity of the differentiated Caco-2 monolayer when exposed to IF-incubated Au NPs [39]. Ag NPs were not included in the Caco-2 assay because of the significant dissolution of Ag NPs, thus the difficulty to exclude the effects of Ag^+^ in the spent media on Caco-2 cells.

Overall, the results demonstrated that even though HA-Au NPs downregulated the transcription of bacteriocin genes in *L. casei*, this did not adversely affect the immunoprotective properties of the bacteria or may have even stimulated the beneficial effects on intestinal epithelial cells.

## 4. Conclusions

Probiotic bacteria are promising candidates as carriers of oral drugs and NPs [67] owing to their ability to resist the gastrointestinal environment. To enable the innovative application of bacteria in nanomedicine, toxicological research of the underlying mechanisms of interactions between the beneficial microbiota and NPs is needed [18]. Elucidation of molecular-level effects of medically relevant NPs on beneficial bacteria facilitates the development of safer and more effective nanomedicines [1]. Preferably, the NP hazard assessment should be performed in physiologically relevant conditions because the standard rich culture media used for bacterial cultivation may underestimate the antibacterial effects. Here, simulated IF was optimized for culturing of human probiotic strain *L. casei*, to enable NP effect assessments on beneficial gut bacteria in the conditions pertinent to the gastrointestinal tract. Future work is needed to further optimize the simulated IF to better support the natural functions of *L. casei* such as bacteriocin production and secretion of other metabolites.

It is important to assess the health risks of the potential nanomedicines already during the developing phase and include the evaluation of their hazards to the human beneficial bacteria; this is crucial because of the unique physiological and ecological characteristics of probiotic bacteria which may make them more vulnerable to NPs than pathogenic bacteria. Specifically, lactobacilli tend to acidify the environment by forming lactic acid as an end product of carbohydrate metabolism which has been shown to affect their susceptibility to Ag NPs due to facilitated Ag^+^ shedding in acidic conditions [44]; this places the commensal bacteria at higher risk than common pathogens which are the intended targets of antibacterial nanomedicines. However, in addition to dissolved Ag^+^, NPs also play a role in the antibacterial action of Ag NPs [68]. Also in this study, not only dissolved Ag^+^ or Au^3+^, but NPs were shown to affect *L. casei*, because the NP solubility in the test media—IF and MRS, was below the antibacterial levels; this stresses the importance of considering the relevance of testing conditions and media composition when assessing the effects of NPs. Au NPs have, in general, no or weak antibacterial effects [69] as was also the case here with *L. casei* and HA-Au NPs. However, both HA-Au and Ag NPs at sub-growth-inhibitory concentrations affected the transcription of bacteriocin genes, suggesting the impact of NPs on the antibacterial activity, and thus, the competitiveness of *L. casei* in the gut microbiota. Still, the immunomodulatory properties of *L. casei* appeared to be either stimulated or unaffected by HA-Au NPs; these results indicated complex metabolic effects of NPs, which should be revealed by future omics, e.g., transcriptomics, proteomics or metabolomics [17,70] assessments of beneficial bacteria.

In summary, the current study demonstrated that NPs with potential usage as nanomedicines can affect the metabolism of a model commensal gut bacterium and potentially influence the functioning of microbiota and immune signaling. Also, since the therapeutic doses of injectable nanomedicines usually span over three orders of magnitude [71], the concentrations of medically relevant NPs assessed in the current study fall within the relevant dosage range, making the results of the study applicable for the preclinical hazard assessment of nanomedicines.

## Figures and Tables

**Table 1:** Primers for real-time qPCR analysis of *L. casei* genes.

Gene Name	Primer (5′–3′)
GAPD	CTTTCCCTGGTGAAGTTAG (F), GTTCAGGAAGTAAGCCATT^®^
LSEI-2386	ATTCATATGGACAGCATCCGTGATGTTTC (F), TTTGAATTCGCTGCCAGAACAAGTTGG^®^(R)
LSEI-2163	AAACATATGAAACGAAAGTGCCCCAAAAC (F), TTTGAATTCGCGACGATCTCTTGAA^®^TC (R)

**Table 2:** Composition of MRS [29] and simulated intestinal fluid (IF). The composition of IF is based on previously published literature [21,22].

MRS	g/L	IF	g/L
Beef extract	8	Meat extract	10.4
Glucose Tween 80	20 1	Glucose Tween 80	10 1.1
K_2_HPO_4_·7H_2_O	2	K_2_HPO_4_	1
CH_3_COONa·H_2_O	5	CH_3_COONa	6
MgSO_4_·7H_2_O	0.2	MgCl_2_·6H_2_O	1
MnSO_4_·4H_2_O	0.05	MnSO_4_·H_2_O	0.01
Triammonium citrate	2	KH_2_PO_4_	1
Oxoid peptone	10	NaCl	0.01
Yeast extract	4	Bile salts	0.1
		Trypsin	100 U/mL

**Table 3:** Hydrodynamic diameters (HDD, Z-average values) and ζ-potentials of nanoparticles in ultrapure water, growth media and “spent” simulated intestinal fluid (IF).

Nanoparticles	HDD (ζ-Potential) in Water, 0 h	HDD (ζ-Potential) in MRS, 0 h	HDD (ζ-Potential) in IF, 0 h	HDD in Water, 24 h	HDD in IF, 24 h	HDD (ζ-Potential) in Spent IF, 24 h
Ag NPs	167 ± 20 nm (−53 ± 2 mV)	573 ± 54 nm (−5 ± 0.5 mV)	497 ± 38 nm (−9 ± 2 mV)	95 ± 2 nm	988 ± 179 nm	557 ± 43 nm (−4 ± 0.6 mV)
HA-Au NPs	55 ± 1 nm (−19 ± 2 mV)	364 ± 65 nm (−3 ± 1 mV)	304 ± 27 nm (−6 ± 1 mV)	20 ± 2 nm	204 ± 10 nm	222 ± 32 nm (−6 ± 1 mV)

The values are means of three replicates ± SD. For HDD, NP samples in the respective media were measured either immediately after mixing with an aqueous medium (0 h) or after 24-h incubation at 37 °C, 200 rpm (24 h).

## Data Availability

The data presented in this study are available on request from the corresponding author.

## Supplementary Materials

The following supporting information can be downloaded at: https://www.mdpi.com/article/10.3390/nano12193377/s1, Table S1: Primers for real-time qPCR analysis of genes in Caco-2 cells, Scheme S1: Schematic representation of the experimental design, Figure S1: Stability of HA-Au NPs in water and modified simulated intestinal fluid (IF) with and without added trypsin, and Figure S2: Antibacterial activity of the spent growth media.
