# Supplementary material for: Silver and Hyaluronic Acid-Coated Gold Nanoparticles Modulate the Metabolism of a Model Human Gut Bacterium Lactobacillus casei
Source: Nanomaterials (Basel). 2022 Sep 27;12(19):3377. doi: 10.3390/nano12193377 (PMC9565723; doi:10.3390/nano12193377)
Supplement: Supplementary file 1 [file nanomaterials-12-03377-s001.zip › nanomaterials-1896983-supplementary.pdf]

Supporting Information

Silver and Hyaluronic Acid-Coated Gold Nanoparticles Modulate the Metabolism of a Model Human Gut Bacterium *Lactobacillus casei*

Wenqian Huang [1,2], Yirong Zhang [1,2], Zhi Li [1,2], Minjie Li [3], Fangfang Li [2,3], Monika Mortimer [2,3,*] and Liang-Hong Guo [2,3,*]

[1] College of Life Science, China Jiliang University, Hangzhou 310018, China; p1909105511@cjlu.edu.cn (W.H.); p1909105520@cjlu.edu.cn (Y.Z.); s20090710032@cjlu.edu.cn (Z.L.)
[2] Institute of Environmental and Health Sciences, China Jiliang University, Hangzhou 310018, China; liff@cjlu.edu.cn
[3] College of Quality and Safety Engineering, China Jiliang University, Hangzhou 310018, China; mjli@cjlu.edu.cn
* Correspondence: mmortimer@cjlu.edu.cn (M.M.); lhguo@cjlu.edu.cn (L.-H.G.)

Table S1. Primers for real-time qPCR analysis of genes in Caco-2 cells.

Gene Name	Primer (5′-3′)
GAPDH	(F) GAAGGTGAAGGTCGGAGTC<br>(R) GAAGATGGTGATGGGATTTC
TNF-α	(F) GTCAGATCATCTTCTCGAACC<br>(R) CAGATAGATGGGCTCATACC
PTGS2	(F) GAGAGATGTATCCTCCCACAGTCA<br>(R) GACCAGGCACCAGACCAAAG
TJP1	(F) GGTGAAGTGAAGACAATG<br>(R) GGTAATATGGTGAAGTTAGAG

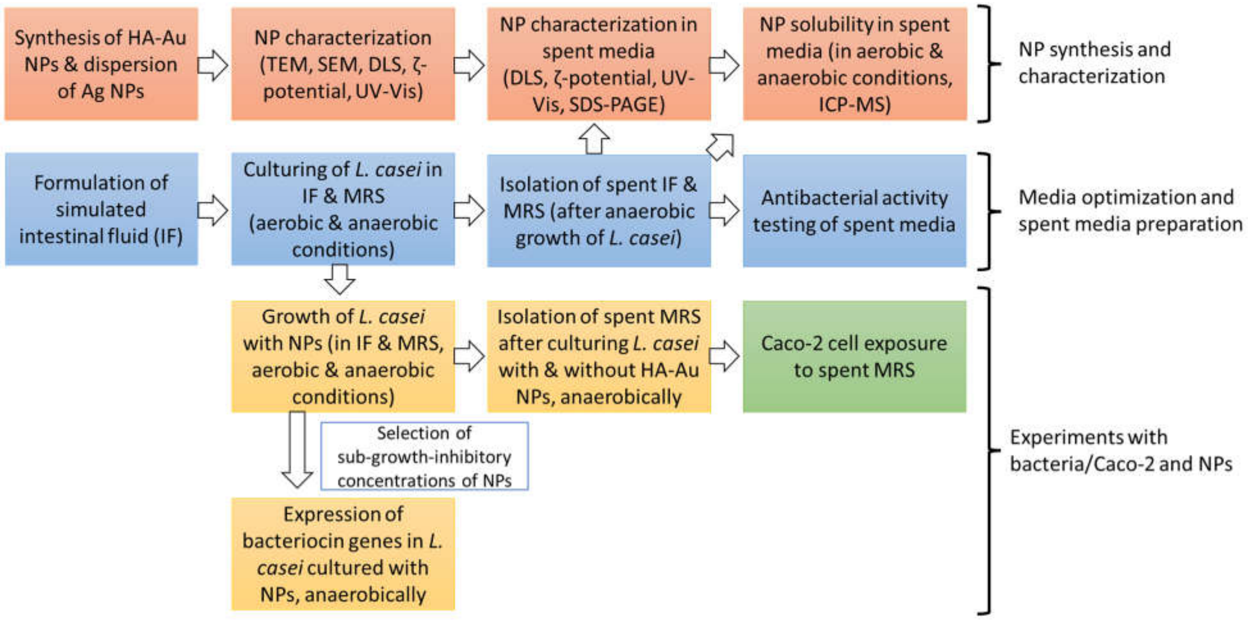

Scheme S1. Schematic representation of the experimental design. NP—nanoparticles, TEM—transmission electron microscopy, SEM—scanning electron microscopy, DLS—dynamic light scattering, UV-Vis—ultraviolet-visible spectroscopy, SDS-PAGE—sodium dodecyl sulfate–polyacrylamide gel electrophoresis, ICP-MS—inductively coupled plasma-mass-spectroscopy, MRS—de Man, Rogosa, and Sharpe broth.

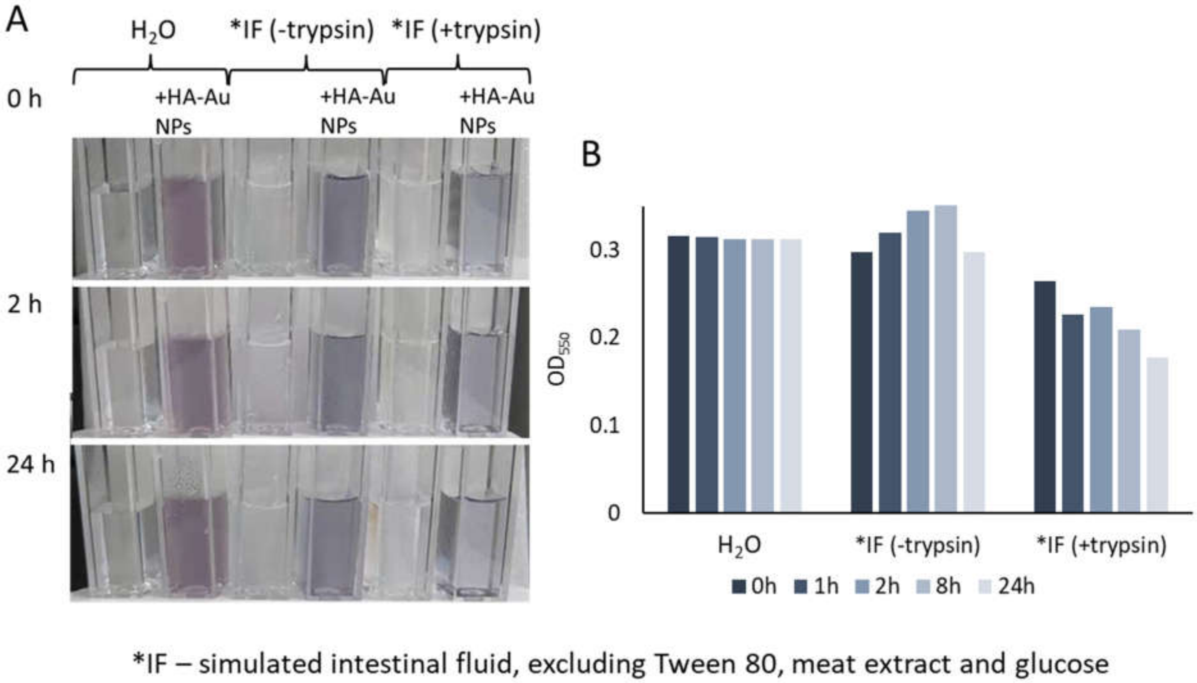

Figure S1. Stability of HA-Au NPs in water and modified simulated intestinal fluid (IF, Table 2) with and without added trypsin (excluding Tween 80, meat extract and glucose, to assess the effect of trypsin on NP dispersion stability). (**A**) Images of cuvettes with aqueous media with and without dispersed HA-Au NPs at 100 mg/L incubated at 37 °C for 0, 2 and 24 h. (**B**) Optical densities at 550 nm (OD_{550}), measured in the cuvettes shown in A, at different time points during incubation at 37 °C. Decrease in OD_{550} reflects the sedimentation of agglomerated HA-Au NPs.

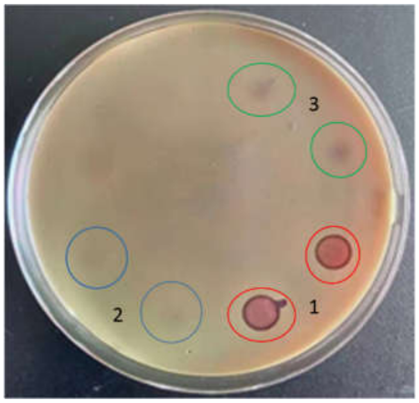

Figure S2. Antibacterial activity of the spent growth media (i.e., media where *L. casei* was cultured). 1—MRS where *L. casei* was cultured for 24 h anaerobically, 2—simulated IF where *L. casei* was cultured for 48 h anaerobically, 3—nisin (100 IU/mL) as a positive control. Columbia blood agar (CBA) plate, spotted with media, was overlayed with the indicator bacterial strain *Micrococcus luteus*. Red agar circles indicate growth inhibition zones of *M. luteus*.
